# Gaining ‘clarity through specificity’ in invited patient participation: A case study of a multifaceted participatory practice in the Netherlands

**DOI:** 10.1111/hsc.13767

**Published:** 2022-03-02

**Authors:** Kasper Kruithof, Clementine Wijkmans, Lotte Ruijter, Janneke Harting

**Affiliations:** ^1^ Department of Public and Occupational Health Amsterdam Public Health Institute Amsterdam UMC University of Amsterdam Amsterdam Netherlands; ^2^ Regional Public Health Service ‘Hart voor Brabant’ 's‐Hertogenbosch Netherlands

**Keywords:** dimensions of participation, invited patient participation, participatory process, rationales for participation

## Abstract

Patient participation is a highly valued principle. Yet, it remains difficult both to achieve it and to assess its added value, as participation is often started without much clarification of what it means or aims to do. In theory, patients may be invited to participate for reasons of democracy, empowerment, knowledge integration and instrumentalism. By making these rationales explicit in a participatory practice in the Netherlands, we aimed to contribute to the long‐needed ‘clarity through specificity’ in participation. Apart from the rationales, our analytic framework included dimensions of the participatory process, reflected by questions like ‘Who participates?’, ‘In what?’ and ‘With how much control?’ We used this framework to conduct and analyse semi‐structured interviews (*n* = 51) with patient participants (20), professionals (14) and researchers (17). We found that the participatory practice included all rationales and that the actual manifestation of an intended rationale very much depended on the design of the dimensions of the participatory process. We conclude that invited participation may gain in clarity by making explicit the rationales for participation. If put at the centre of attention, and made the leading factor in the design of the dimensions of the participatory process, explicit rationales may support the realisation of participation in practice and prevent it from resulting in mere window‐dressing.

**What is known about the topic**

- Invited patient participation, i.e. participation arranged by an external agency, is often difficult to realise due to a lack of conceptual clarity.
- Theorists have argued that making explicit the rationales for participation or the dimensions of the participatory process would improve conceptual clarity.
- Empirical studies that apply these rationales, dimensions or both are rare.

**What this paper adds**

- Clarity in invited participation may be improved by making explicit the rationales for participation and the dimensions of the participatory process.
- An analytical framework including both these rationales and dimensions can help to elucidate their interdependency in invited participation.
- Applying such an analytical framework may also improve the realisation of invited patient participation in real‐life practice.

## 1 INTRODUCTION

In public health, it is widely believed that patient participation contributes to both participants’ welfare and health outcomes (Domecq et al., 2014). Consequently, both in the Netherlands and in other countries, patients are increasingly being involved in health services, policy and research (Redman et al., 2021; Teunissen & Abma, 2013; van de Bovenkamp et al., 2010). Their involvement includes, for example, assessing health care quality, setting national health policy agendas and participating as co‐researchers. While there is some evidence to support the added value of patient participation, explaining or predicting contributions arising from participation remains difficult (Domecq et al., 2014; Nitsch et al., 2013; Rifkin, 2014). The main explanation for this difficulty is that invited participation, i.e., patient participation arranged by an external agency, is mostly started without much clarification of what it means or aims to do (Cornwall, 2008; Kenny et al., 2014). Such an unspecified approach may cause ambiguities regarding participation purposes and approaches (Morgan, 2001), hamper participation processes and outcomes (Morgan, 2001; Rifkin, 2014), and complicate the interpretation of evaluation studies (Conklin et al., 2015; Rifkin, 2014). That participation is in need of 'clarity through specificity' was noted 40 years ago (Cohen & Uphoff, 1980), while present‐day reviews concluded that since then not much progress has been made (Boote et al., 2002; Cornwall, 2008; Rifkin, 2014).

Theorists have argued that making explicit the different underlying reasons—or rationales—for participation would be advantageous in this respect (Cohen & Uphoff, 1980; Stirling, 2008). For instance, patients may be invited to participate for reasons of democracy (normative rationale), empowerment (transformative rationale), knowledge integration (substantive rationale) or efficiency (instrumental rationale) (Stirling, 2006; White, 1996). A related argument suggests that the manifestation of any rationale for participation would strongly depend on the design of the participatory process (Cohen & Uphoff, 1980; Cornwall, 2008; Farrington et al., 1993). Here, ‘design’ refers to the dimensions of participation, mirrored by questions like: ‘Who participates?’, ‘In what?’ and ‘With how much control?’ (Cornwall, 2008). So far, practical applications of rationales and dimensions have been scarce (Stirling, 2006; Wesselink et al., 2011). We are aware of three studies that used either rationales (Glimmerveen et al., 2018; Wesselink et al., 2011) or dimensions (Lewis et al., 2019) to disentangle participatory practices. These studies noticed, for instance, that involving citizens in a local professional organisation occurred for possibly incongruent reasons (i.e. normative and instrumental) (Glimmerveen et al., 2018), and that involving residents in place‐based community initiatives required attention for two potentially conflicting dimensions, i.e. the breadth (Who participates?) and the depth (With how much control?) of participation (Lewis et al., 2019). For both rationales and dimensions separately, it was concluded that their use could help to better understand real‐world participation (Lewis et al., 2019; Wesselink et al., 2011). Based on these conclusions and previous suggestions (Cohen & Uphoff, 1980; Cornwall, 2008), we propose that the combined application of rationales and dimensions would be most effective in gaining ‘clarity through specificity’ in participation. Hence, such an approach could further help to colour in the ‘mosaic’ that was used as the metaphor to reflect the variety and dynamics of participation in practice (Tritter & McCallum, 2006).

To explore the added value of combining rationales and dimensions in unravelling participation, we made use of both to examine a multifaceted participatory practice in the Netherlands: the civil society organisation Q‐support (www.q‐support.nu). Q‐support was established with the aim to improve the situation of Q fever patients, by offering individual advice, organising collective support and inviting tenders for research (Q‐support, undated). From the start, Q‐support aimed to involve the perspectives of Q fever patients in its operational processes through organised participation, thereby becoming an ‘invited space’ for participation (Cornwall, 2008). Participation was interpreted as the involvement of patients in three decision‐making contexts (Charles & DeMaio, 1993; Fredriksson & Tritter, 2017): that of service delivery, policy development and scientific research. For its participatory approach, Q‐support was awarded as a ‘best practice’ by the Netherlands Organisation for Health Research and Development. Supported by this recognition, we selected Q‐support, with its different participatory processes in different decision‐making contexts, as the subject of our study. For more background information, see Box 1.

BOX 1 **Background information on Q fever and Q‐support**

**Table:** 

*Q fever*
Q fever is a zoonosis: an infection caused by a bacterium that is transmitted to humans by goats and sheep (Dijkstra et al., 2012). Although 90% of the patients with an acute Q fever infection recover, 1%–5% develop chronic Q fever (CQ) and 20% suffer from post‐infection Q fever fatigue syndrome (QFS) (Dijkstra et al., 2012; Morroy et al., 2016).
*Q‐support*
Q‐support was set up with a subsidy of 10 million Euro that the Dutch Ministry of Health, Welfare and Sports had made available in response to two critical reports about the national government's reaction to the latest Q fever epidemic in the Netherlands (2007–2009) (Evaluation Committee Q fever, 2010; Van der Bijl et al., 2012). In this epidemic, between 50,000 and 100,000 people got infected, thousands developed acute Q fever and at least 74 died from the disease (Dijkstra et al., 2012). Q‐support was funded for a period of five years (2013–2018) to improve the situation of Q fever patients, especially of those with CQ and QFS, by offering individual advice, organizing collective support and inviting tenders for research (Q‐support, undated).
*Dispute*
Although most Q fever patients saw the foundation of Q‐support as a necessary investment to improve their situation, they typically did not regard it as a sufficient response to the neglect they had experienced during the Q fever epidemic. Many Q fever patients also desired financial compensation for the physical, financial and emotional distress the epidemic had caused them. At the time of the study, two legal claims about compensation were in progress: one against the State of the Netherlands and one against about 100 goat farmers. However, these claims were not supported by Q‐support, as they were considered incompatible with the organization's governmental remit (Interviews and documents collected for this study).

With the ultimate aim to contribute to clarity in patient participation, we aimed to answer the following research questions about Q‐support: ‘Which rationales for participation were intended and became manifest?’, ‘How were the manifested rationales related to the dimensions of the participatory process?’ and ‘What other conditions were of influence?’ In doing so, we explicitly intended to learn from—and not to judge—the manifestations of participation and the participatory processes through which these manifestations came about.

## 2 METHODS

### 2.1 Design

We examined the participatory practice of Q‐support in a case study (Bryman, 2016; Yin, 2003). We studied the organisation's three decision‐making contexts (from here on ‘trajectories’). Q fever patients were involved as experiential experts in service delivery (the Care trajectory), as representatives in policy development (the Policy trajectory), and as assessors and monitors of scientific research (the Research trajectory). Our study was both deductive, by applying the theory‐based rationales and dimensions to the empirical case of Q‐support, and inductive, by making comparisons of the participants’ experiences within and between the three participatory trajectories in Q‐support, in order to contribute to further theory development (Bryman, 2016).

### 2.2 Respondent selection

First, KK and JH familiarised themselves with the case through orientating talks with the managing director of Q‐support and the chair of the Scientific Committee, and through a group discussion with seven patient participants. Q‐support also gave us access to its internal documentation, including policy documents, process descriptions and minutes of meetings. Based on this information, we invited all patient participants, professionals and researchers that were or had been involved in one of the three participatory trajectories within Q‐support (*N* = 53), for an interview. We chose such exhaustive sampling because we expected different experiences for the different types of participants and the different participatory trajectories. Two persons declined our invitation: one patient participant felt too sick for an interview and one researcher felt insufficiently involved in the case.

### 2.3 Sample

The final sample (*N* = 51) included 20 patient participants, 14 professionals and 17 researchers.

Thirteen patients were involved in one participatory trajectory: two experiential experts in the Care trajectory, five members of the Think Tank in the Policy trajectory and six relevance assessors of research proposals in the Research trajectory. Three patients were involved in more than one trajectory and four had another role. The patient participants were between 29 and 75 years old. Ten were female, 10 male. Their education level varied.

The professionals included the managing director of Q‐support, the chair of the Patient Committee, the chair of the Scientific Committee, the chair of the Think Tank, the Board secretary, the management secretary, the project manager for patient facilities, the intake coordinator, the programme manager and three process managers of the Care trajectory, the project manager for work and income, and the instructor of a coping‐with‐disease course for Q fever patients. The sample of professionals consisted of 10 females and 4 males. All of them were highly educated.

All researchers were involved in one or more studies funded by Q‐support. Ten of them were female and 7 male. They were all highly educated.

### 2.4 Data collection

Between August 2017 and December 2017, KK interviewed all respondents face‐to‐face in a semi‐structured way (Bryman, 2016).

The main themes addressed were the intended rationales for participation, the rationales that became evident, the dimensions of the participatory process and other conditions influencing the participation. We used prompts to encourage respondents to not only reflect on their actual experiences, but on their ideal of patient participation as well. Examples of such questions were: How would your ideal of patient participation look like? Which patient participants should be involved, and why? What further factors would be important to reach your ideal? More context‐specific questions addressed the course of events and the specification of tensions in the participatory process.

The interviews were held at the place the respondent preferred: at home (patient participants), at the office of Q‐support (patient participants and professionals) or at the university (researchers). Interviews took between 40 and 100 min each, with a mean of one hour.

### 2.5 Data analysis

The interviews were transcribed verbatim and analysed in a qualitative data analysis programme (MaxQDA, 2016), applying grounded theory procedures and techniques (Strauss & Corbin, 1990) (Bryman, 2016). Rich interviews (Ogden & Cornwell, 2010) were coded first (*N* = 34), until saturation was reached. Less rich interviews were coded next (*N* = 10) and confirmed our initial findings. The least informative interviews were checked for contradictory evidence (*N* = 7), which was not found.

The coding started with theory‐based core categories related to the rationales for participation and the specification of the dimensions of the participatory process. In addition, open coding was used to identify other conditions facilitating or hampering the manifestation of rationales for participation in practice. To identify relations between rationales, dimensions and conditions, we compared respondents within one participatory trajectory. To identify recurrent patterns—if any—we compared these relationships across the three participatory trajectories.

To optimise the reliability of our analysis, the first three interviews, one with a patient participant, one with a professional and one with a researcher, were independently coded by KK, LR and JH using the same core categories. This procedure was repeated by KK and JH for another three interviews. Discrepancies made us split up the codes for rationales into separate codes for those that were intended and those that became evident in practice. Relations between rationales, dimensions and conditions, as well as patterns in these relationships, were extensively discussed between KK and JH, and finally agreed upon by all authors.

To optimise the internal validity of our study, member checks were held with the managing director of Q‐support, the chair of the Scientific Committee and a group of eight patient participants who covered the three participatory trajectories within Q‐support. Topics discussed were the design of the study, the analysis of the interviews and the initial results.

### 2.6 Ethical considerations

According to the Dutch Medical Research Involving Human Subjects Act, this study did not require approval by a medical research ethics committee. Respondents were informed, by email and orally, about the aims and methods of the study, and about their rights, including the right to withdraw from the study, and given the opportunity to ask questions. Verbal consent was obtained and recorded. To protect the anonymity of our respondents, we refer to them as patient participant, professional or researcher when reporting our findings. If the content of a quotation still precluded full anonymity, respondents were asked permission for publication by email.

## 3 FINDINGS

The findings are reported for each decision‐making context separately. In the main text, we illustrate the findings with quotes from ten different respondents, including patient participants (*n* = 4), professionals (*n* = 3) and researchers (*n* = 3). For an overview of, and supporting quotes for, the interdependencies between rationales, dimensions and other conditions, see Table 1.

**TABLE 1:** Role of dimensions of participation and other conditions in the manifestation of rationales in three decision‐making contexts

Intended rationale	Manifested rationale	Dimensions/Conditions	Quotations supporting dimensions/conditions
Care trajectory			
*Transformative* For newly enroled patients seeking advice and support, through increased recognition and empowerment For patients acting as experiential experts, through personal empowerment	*Transformative* As intended [according to patient participants and professionals]	*Who participates?* Patients who are able to rise above their own problems and anger, are able to put newly enroled patients first, and know when and how to make use of their own story [according to patient participants and professionals] *Conditions* Clear and accepted role division between experiential experts (offering recognition through proximity) and professionals (directing the intake process and guarding its boundaries) [according to patient participants and professionals]	“The anger I feel towards the [social welfare institution], I will never communicate that anger as an experiential expert. No, I can tell them which steps they can take, advise them. But I'm not going to tell them all they've got coming. That will dishearten them.” [Patient participant] “You should be able to listen carefully and also be prepared to express your own feelings, I think. […] Well, I'm really an open book. But you also have to be careful you're not just telling your own story. You have to listen very carefully and ask the right questions.” [Patient participant] “There is also a guilty party [i.e. the state] you can point the finger at, you see. […] On the one hand, of course, we're being funded by it [the state]. On the other hand it [this anger] is part of their illness and all the consequences that come along with it, like losing their job and income and so on. […] In the beginning I found that very difficult. To acknowledge those patients [experiential experts] in the problems they experience, but also to maintain a distance from them.” [Professional]
	*Substantive* For newly enroled patients seeking advice and support For the Q fever patient population as a whole [according to professionals]	*Conditions* Professionals who collect relevant information that emerges during the intake sessions, who tailor their advice and support for individual patients, and pass on the information to the management [according to professionals]	“It's often the case that a [newly enrolled] patient gets a sense of connectedness with the experiential experts. And from that feeling of connectedness, the process manager gets a lot of information.” [Professional] “Based on the intakes, based on the intake reports, the process manager comes to me, like, I have this case… and can we do something about it?” [Professional]
Policy trajectory			
*Normative* For the Q fever population as a whole, through involving a representative sample *Substantive* For the Q fever population as a whole, through better tailored policies	*Nominal* For both patient members in the panel and the Q fever patient population as a whole [according to most patient participants, some professionals and the management] *Substantive* For the Q fever patient population only on some topics [according to some patient participants, some professionals]	*Who participates?* Patients who are—or are not—able to rise above their own problems, who have professional skills to work and discuss matters within the organisation [according to some patient participants, and to professionals and management] *Participation in what?* Patient's opinions that do—or do not—fit the scope of the organisation [according to professionals and minority of patient participants] *With how much control?* Uncertainty—or clarity—about the degree of decisional power of the panel [according to patient participants and management] Lack of decisional power of the panel [according to most patient participants] *Conditions* Communication and feedback on contributions of the panel—or the lack thereof—from the organisation's management [according to most patient participants]	“If the Think Tank is intended to provide us with advice, this outcome is particularly meagre. There may be several causes. Patients […] may not be familiar with the matter and may lack a comprehensive view, may be [focused] too much on control of what we do, may be stuck too much in their own opinions or experiences.” [Internal evaluation report Work and Income] “The Think Tank people were critical and that's okay, but we hadn't found the right format, we hadn't managed to achieve cooperation. […] So in the end I felt some friction, also in the rest of the management team, like: “We don't want to attend this Think Tank anymore.” Because they had the feeling that they were always facing some kind of forum or tribunal.” [Professional] “I think that the Think Tank people sometimes raised questions [e.g., about the financial compensation] that were beyond the framework, and then it's difficult.” [Patient participant] “Well you know, the people in the Think Tank expected to be able to fully intervene in the policy, but it's still an advisory body, not a decisional body. That was more or less the key problem.” [Professional] “And if you don't get the answer from the director […], even if the answer is “No” […], then people don't feel they're being heard. So in the end I think the Think Tank has been sent from pillar to post, and they feel they haven't been taken seriously.” [Patient participant]
	*Substantive* For the Q fever patient population as a whole [according to some patient participants and some professionals]	*Who participates?* Patients who are informed and sufficiently empowered to take matters in their own hands [according to some patient participants] *Conditions* Searching for and creating opportunities to exert influence outside of the organisation [according to some patient participants]	“Something was presented. That gives you some insights into the minister's goals. And that made me start a campaign with some others to send a lot of letters to the Ministry. […] But that's an individual action. […] Q‐support isn't an activist organization. But I do have that sense of action in me. And I do determine the moment to act, based on the knowledge that I’ve gained from the Policy Trajectory.” [Patient participant]
	*Transformative* For all patient members in the panel: professional and/or personal growth [according to all patient participants]	*No specific dimension/condition emerged from the analysis*	—
Research trajectory			
*Substantive* For the Q fever population as a whole, through realising research of higher relevance	*Substantive* As intended in the relevance assessment of research proposals [according to most patient participants, most professionals and some researchers]	*Who participates?* Patients who have a broader perspective on Q fever than their own clinical picture [according to most patient participants and professionals] Higher educated patients who are able to understand research proposals [according to most professionals and researchers] *With how much of control?* Clearly defined decisional power for patients acting as relevance assessors [according to all patient participants and professionals] *Conditions* Clarity about role division between patients (having a say on relevance) and professionals (having a say on quality) [according to all patient participants and professionals] Research proposals written in language that patients can understand [according to most researchers and professionals]	“Patients seeing the relevance for the larger body of patients, and the relevance for future Q fever patients” [Researchers] “They must be able to read a research proposal, right. So you expect a certain level. You have to write it in fairly easy terms. But still that's not for everyone. You can only grasp what could be the results if you have a certain level of reading. […] It's not for everyone.” [Researcher] “That has to do with the framework being very clear. That's what the patients really looked at, well, the patients decide which research project will be funded, based on relevance. And if the patients really don't like it, even if it is the very best research, then it won't happen. So there you see that the framework must be clear.” [Patient participant]
	*Instrumental* For researchers, through improvements in the research process due to the monitoring of study progress [according to some patient participants and some researchers]	*Who participates?* Average patients who can counteract scientific biases [according to most researchers] *Conditions* Research reports written in language that patients can understand [according to most researchers and professionals]	“The preference is not: as highly educated as possible. That sounds nice because it makes it easy to convince them. But the risk, also from our own experience, is that you then quickly fall back on your own scientific way of thinking, and that the participant goes along with that. While what we want to get is: “No, I don't recognize this,” or “I do recognize that.” So perhaps a low educational level is actually better.” [Researcher]
	*Nominal* For patients monitoring study progress and researchers responding to the patients’ comments [according to most patient participants and most researchers]	*Who participates?* Patients who understand—or not—how research is conducted and what are the peculiarities of the research process [according to most patient participants, most researchers and most professionals]	“They [the patients] sometimes asked: “What's going on?” Then I sometimes said: “Yes well, that's the way the research world works, that's what you can expect.” So I influenced them to some extent by giving them more insights into the process of doing research. Because sometimes they had, like,… they'd say: “Pull the plug”, or “We had expected some results by now.” And then I’d say: “Yes, but that's not possible.”” [Professional]
	*Transformative* For patient participants: empowered by playing an active acting role in finding solutions for Q fever‐related problems [according to all patient participants]	*Conditions* Being acknowledged as an experiential expert who provides valuable knowledge [according to most patient participants]	“If you are allowed to think along and make use of your personal knowledge, well, I think this has also positively influenced my process. That you can talk along […], because that is the experience […], I thought it was taken seriously.” [Patient participant]

### 3.1 Care trajectory

#### 3.1.1 Introduction

In the Care trajectory, individual Q fever patients could apply for personal advice and support regarding the treatment of the disease and its consequences for their daily lives, their work and financial situation, and their wellbeing and societal participation. Newly enroled patients started off with an intake session jointly led by a professional process manager and a patient participant as experiential expert. During these intakes the process manager was responsible for setting the boundaries of the interview and for composing an individually tailored support programme. Experiential experts were either self‐selected or invited by the management.

#### 3.1.2 Intended rationale

The intended rationale for additionally involving another Q fever patient in the intake as an experiential expert was transformative, i.e. empowering new patients by offering ‘recognition through proximity’, since many felt ignored by traditional care agencies.

#### 3.1.3 Manifested rationales

The employment of an experiential expert did indeed contribute to transformative outcomes. Being recognised for what one is going through by meeting another Q fever patient who had gone through a similar process was perceived as empowering for newly enroled Q fever patients.

*So you act as an example: "Oh, he [the experiential expert] is also … he has it too, but he still does things, even if he’s tired. He has found his way.” […] They [the newly enrolled Q‐fever patients] also feel very bad, yes, ignored, angry, and then they get an example showing: "Yes, despite that … you can still make something of your life."* [Professional]

Employing an experiential expert led to substantive outcomes as well. The ‘recognition through proximity’ led to a more open intake situation in which newly enroled patients were likely to share more personal stories. This created a better understanding of the problems the patient experienced and, therefore, better opportunities to tailor the support programme to their individual needs.

*It’s often the case that a patient gets a sense of connectedness with the experiential experts*. *And from that feeling of connectedness in the conversation, the process manager gets a lot of information*. [Professional]

Getting a better understanding of an individual patient's problems also enabled Q‐support to adapt its support programmes to the Q‐fever patient population as whole, e.g., with regard to the problems associated with work and income that many patients expressed during the intake interview.

*Based on the intakes, based on the intake reports, the process manager comes to me, like, I have this case… and can we do something about it?* [Professional]

Finally, the Care trajectory had a transformative outcome for the patient participants themselves: by performing the role of experiential expert they felt themselves grow personally and/or professionally.

*I became aware of qualities that I didn’t know I had. […] The whole process at Q‐support has given me a more positive outlook towards the future, despite my symptoms*. [Patient participant]

#### 3.1.4 Dimensions

‘Who participates?’ was seen as the most important dimension for the transformative and substantive rationales to become manifest. According to both the process managers and the patient participant, experiential experts should be able to transcend their own situation, rise above their own anger and problems, and put the new clients’ needs first. In addition, it was seen as important that experiential experts knew when and how to make use of their own story.

#### 3.1.5 Other conditions

For transformative outcomes, a clear and mutually accepted role division between the experiential expert and the process manager was seen as important. Here, difficulties arose with regard to the claim against the state by Q‐fever patients. The anger that experiential experts felt towards the government, and associated activism, did not fit in with the aim of Q‐support, funded and founded as it was by the same government. Important for the manifestation of substantive outcomes was sufficient responsiveness of the organisation's management.

### 3.2 Policy trajectory

#### 3.2.1 Introduction

In the Policy trajectory, Q fever patients participated in a Think Tank: a patient panel that advised the management of Q‐support about the organisation's policies. The patients’ advice related to the spending of resources on three different policy themes: Body & Mind, Work & Income, and Public Awareness. The management invited patients to become a member of the Think Tank.

#### 3.2.2 Intended rationales

The management aimed to include a representative sample in the Think Tank, as participation in policy decisions was seen as a normative right. At the same time, Q‐support also expected participation to create substantive outcomes in terms of tailoring the policies and activities of the organisation to the patient populations’ problems and needs.

*Well, of course you get an amount of money, but what are the patients’ needs? […] How can you find out better than by involving the patients and talking with them, something like a partnership. It’s from this idea that the Think Tank emerged*. [Patient participant]

#### 3.2.3 Manifested rationales

A small group of patients felt they had indeed made substantive contributions within the Think Tank. They were able to put certain topics, mostly related to work and income, on the agenda of the organisation, and felt taken seriously by the management.

*The feeling, in any case, that I’ve been able to make a contribution, to help people, to help influence processes, contributing initiatives that have been adopted by [the management], on behalf of Q‐support*. [Patient participant]

However, the majority of the patients felt that their participation had resulted only in nominal outcomes. They did not have the impression that their recommendations, e.g., about the position of Q‐support regarding the claim for financial compensation for Q fever patients by the government, were being used, and they spoke of insufficient reactions by the Q‐support management.

*The idea is great. What we tell them is great. But very little is done with it*. [Patient participant]

Even though most patients were dissatisfied with their role, they too reported transformative outcomes for themselves in terms of ‘growth’: they ‘had grown’ professionally and/or in the way they coped with their situation as a person. Moreover, some patients looked for other ways to exert a substantive influence. Using the knowhow they had gained through their Think Tank membership, they started to approach stakeholders outside Q‐support about issues that did not fit the scope of the organisation.

*Something was presented [at the Think Tank]. That gives you some insights into the minister’s goals. And that made me start a campaign with some others to send a lot of letters to the Ministry. […] But that’s an individual action. […] Q‐support isn’t an activist organization. But I do have that sense of action in me. And I do determine the moment to act, based on the knowledge that I’ve gained from the Think Tank*. [Patient participant]

#### 3.2.4 Dimensions

The members of the Think Tank had an unclear role: whereas they themselves felt that they should have actual decisional power, the management felt that they should only give advice. This uncertainty—or disagreement—about the decisional power undermined a substantive contribution.

The lack of substantive participation also had to do with ‘Who participates?’. In view of the normative purpose of the Think Tank, the management had invited patients with different characteristics as to represent various clinical pictures and opinions. Inherent to this diversity, the Think Tank also included patients with opinions that did not fit the scope of Q‐support. Therefore, the management regarded them as being too activist and their advice as being of little use, which hampered the manifestation of the substantive rationale. Apart from the management, also some members thought that other patients in the Think Tank were too activist, and that this was counterproductive.

Another consequence of the heterogeneous composition of the Think Tank was a perceived lack of professional qualities. Some patients were criticized by professionals for not being capable of rising above their own problems and not being able to represent broader issues and the Q fever patient population as a whole.

#### 3.2.5 Other conditions

Members of the Think Tank underlined the necessity of communication in the Policy trajectory. They spoke of a lack of feedback from the management on the status of topics they wanted to act on. Although they understood that their recommendations could not always lead to concrete action, they would have appreciated regular updates on what was or was not done with their efforts.

### 3.3 Research trajectory

#### 3.3.1 Introduction

In the Research trajectory, Q fever patients assessed the relevance of the proposals submitted in response to Q‐support's tenders for research, while the quality was assessed by a professional Scientific Committee. As a rule, proposals without a sufficient relevance score were not funded by Q‐support, regardless of their scientific quality. Patients also monitored the progress of the research by evaluating the interim study reports and going through a question‐and‐answer procedure with the researchers.

#### 3.3.2 Intended rationale

The main rationale for participation in the Research trajectory was substantive. The involvement of patients in appraising the relevance of research proposals was assumed to improve the quality and significance of the knowledge produced, i.e., the study outcomes.

*Q‐support […] wants to connect the world of the patient with that of the researcher. With the aim of ensuring that researchers and patients find each other easily and without reservations. Based on the conviction that this yields better and more relevant research*. **[**Internal Participation Report, 2016]

The monitoring of the progress of the studies was also intended to substantively influence research efforts in terms of direction or approach. Patients had to apply for a position in the Research trajectory, except for the few with a scientific background who were invited by the management.

#### 3.3.3 Manifested rationales

The Q‐fever patients’ appraisals of the relevance of the research proposals—and the weight attached to their appraisals—yielded two examples of substantive participation in terms of funding being allocated to different research than would have been the case without the patients’ relevance scores. One research proposal regarding the impact of Q fever on work‐related and psychosocial problems was funded—despite being given a low quality score by professionals—because the patients gave it a high score for relevance. Another research proposal, on the cost‐effectiveness of a treatment for Q fever, was not funded—despite being given a high quality score by professionals—because the patients gave it a low score for relevance.

*That request was shot down mercilessly by the patients in the first round, because they said: "Costs, costs, what does it matter how much it costs?"* [Researcher]

In the patients’ monitoring of the progress of the studies, the substantive rationale hardly became manifest. Rather than influencing the direction of the research, the comments given on the interim study reports mostly resulted in instrumental outcomes, if any. For instance, patient participants enabled some researchers to include a more diverse sample of patients in their study.

*That it’s very important for our research to find as wide a range of people as possible. And the kind of language that we use… well, you often stay in your own little bubble. […] And if you ask patients to think along, you might come up with other ideas [for recruitment].”* [Researcher]

Despite these instrumental outcomes, the patients’ monitoring of the study process often resulted in nominal participation, as the researchers were frequently dissatisfied with the—from their perspectives—irrelevant questions.

*When they ask: “what’s an antibody?” […], then I don’t really think that I have to answer. […] You can Google what’s an antibody. […] It literally takes [me] hours. […] Time that I could have spent on something else, and I would almost go so far as to say, spent more usefully*. [Researcher]

Finally, patients also reported transformative outcomes, such as feeling enabled to accept and improve their situation by being involved in the search for possible future cures for their disease.

#### 3.3.4 Dimensions

Regarding the appraisal of the relevance of research proposals, the level of clarity regarding both the role and the decisional power of the patient participants was named as an important dimension for substantive outcomes.

With regard to the dimension of ‘Who participates’, the management of Q‐support selected relatively highly educated patients, with the idea that they could understand the research projects better and therefore make stronger substantive contributions. While patients thought that a broader view on Q fever than their own clinical picture should be enough, some researchers agreed with the management about the selection of higher educated patients.

Regarding the monitoring of the progress of the studies, most researchers advocated the involvement of average patients, both for substantive and instrumental reasons, as it was exactly those patients who could counteract scientific biases and improve the readability of the study reports.

#### 3.3.5 Other conditions

An important prerequisite for the Research trajectory was that both research proposals and progress reports were written in language that was understandable for patients. As another condition, patients had to have or gain sufficient understanding of the scientific process, especially of the time such a process requires. Because of the long running time of most studies, patient participants came to feel that researchers were out for personal success and therefore not ‘in it for the right reasons’, i.e. solutions for the problems they experienced.

## 4 DISCUSSION

### 4.1 Summary

In this case study, we looked at one organisation—Q‐support—which involved three decision‐making contexts. We examined which rationales for participation became evident and how these manifestations were related to the dimensions of participation and to other conditions. We saw that all rationales we had identified in the literature became manifest in the organisation's invited participation practice, and that different rationales were involved in each of the decision‐making contexts. Whether or not a particular rationale became manifest very much depended on the design of the dimensions of the participatory process. Some rationales could become manifest simultaneously, provided that their intended outcomes did not conflict. Other combinations were problematic, because the rationales involved required a different design of one and the same dimension. The power dimension appeared crucial for some, but not for all rationales to become manifest. Other dimensions for manifestation varied across rationales, but a common requirement was a tightly organised participatory process, including clarity about the scope and procedures, well‐defined roles and tasks for all involved, and regular feedback from the inviting organisation about the patients’ contributions.

### 4.2 Interpretation

While a relationship between the rationale for participation and the dimensions of the participatory process has been put forward before (Cohen & Uphoff, 1980; Cornwall, 2008; Wesselink et al., 2011), our study is the first to offer an empirical basis for their interdependency. In the Care trajectory, for example, the design of the ‘who’ dimension was crucial for the manifestation of the transformative rationale, while that of the ‘power’ dimension hardly played a role. By contrast, in the Research trajectory, the design of the ‘power’ dimension was decisive for the manifestation of the substantive rationale, while that of the ‘who’ dimension was more flexible. These findings indicate that a misalignment between rationale and dimensions may further explain why invited patient participation often results in merely nominal outcomes (e.g. Domecq et al., 2014; Zakus & Lysack, 1998), especially as rationales in participatory practice often remain implicit (Cornwall, 2008). Our study thereby substantiates the asserted importance of ‘intentionality’ (Stirling, 2008, p. 268). Hence, we agree that the rationale for invited participation should be made explicit (Cornwall, 2008; Wesselink et al., 2011) and recommend to give it a leading role in the design of the dimensions and the choice of methods.

We found that the simultaneous manifestation of different rationales appeared to be problematic when each asked for a different design of the ‘who’ dimension. For example, in the Policy trajectory, the representative sample of participants, selected to achieve normative participation, brought—in the perspective of the organisation—along opinions that did not sufficiently fit the organisation's scope to simultaneously realise substantive participation. The presence of multiple rationales in a single participatory practice is not uncommon and may create a confusing empirical reality (Cornwall, 2008; Glimmerveen et al., 2018; Stirling, 2006). If one specific dimension requires to be differently designed for their simultaneously manifestation, none of the rationales present may succeed in becoming manifest (Glimmerveen et al., 2018). Therefore, apart from making rationales explicit (Cornwall, 2008; Wesselink et al., 2011), additionally acknowledging conflicts between rationales and the required design of the dimensions may be of vital importance too for the success of invited participation.

We found that the transfer of decisional power to patients was especially important for a substantive rationale to become manifest. Whereas in the Policy trajectory, the manifestation of this specific rationale was hampered by lack of clarity about decisional power, in the Research trajectory its manifestation was facilitated by the binding mandate given to patients to decide on the relevance of the research proposals. Our results support the assertion that the quality of knowledge, which is key to the substantive rationale, is not a value‐free phenomenon, but influenced by the political context (Stirling, 2006). In the Policy trajectory, we recognised such a political influence in the—sometimes implicit, sometimes explicit—delineation of the scope of the ‘participatory playing field’ through the governmental funding of the Q‐support organisation. Whereas patients who agreed with the boundaries of the ‘playing field’ felt that they did make substantive contributions, those who wanted to have a say on issues outside of this ‘playing field’ perceived their contribution as nominal. This seems to equal the ‘paradox of expert patient participation’ (Wilson et al., 2007): patients who are deemed expert enough to participate from an substantive perspective are those who tend to agree with the prevailing medical paradigm. We therefore argue that the substantive rationale may only become manifest if the boundaries of the ‘participatory playing field’, i.e., the answers to the questions ‘Participation in what?’ and ‘With how much control’ (Cornwall, 2008), are agreed upon by all stakeholders. Deliberative approaches could be helpful in this respect (Abelson et al., 2003).

Finally, we saw how some rationales easily became manifest simultaneously. This happened only if their intended outcomes were perceived as non‐conflicting—or even as congruent—by the stakeholders involved, and if the design of the dimensions supported both their manifestation. In the Care trajectory, for instance, the employment of experiential experts, who were able to transcend their own situation (Toikko, 2016) and put the new clients’ needs first (the ‘who’ dimension), enabled the transformative as well as the substantive rationales to become manifest, as both were expected—and perceived—to improve the situation of individual patients and the Q fever patient population as a whole. We agree that patient participation may result in effects that were never envisioned at the outset (Cornwall, 2008; White, 1996), as we saw such an unexpected development in the Policy trajectory. However, our findings may also imply that a better understanding of the possible interactions between rationales and dimensions, as the building blocks of invited participation, could perhaps help to envision—or even support—initially unexpected outcomes (Boote et al., 2002). We think that our approach, in which we combined complementary frameworks for participation (Cohen & Uphoff, 1980; Farrington et al., 1993; White, 1996), may offer added value in this respect.

### 4.3 Strengths and limitations

One strength of our study is that the Q‐support case included three decision‐making contexts, each starting from a different rationale and using a differently designed participatory process. This allowed us to describe a greater variety of rationales than other empirical studies, which included single participatory practices dominated by the instrumental rationale (Domecq et al., 2014; Wesselink et al., 2011). Our multifaceted case also allowed us to discover relations between rationales and dimensions as a first step towards identifying patterns.

A second strength was that our framework included rationales for participation that in essence differed from each other. This comprehensiveness created the opportunity to distinguish between rationales that otherwise easily get mixed up (Stirling, 2006, 2008). For instance, while instrumental participation without transfer of power to participants tends to be valued as nominal (Stirling, 2006), our more subtle inference was that this depends on whether the intended outcomes for the different stakeholders align or conflict.

A first limitation is our single case study design. Although we identified several relations between rationales and dimensions, from a single case it remains problematic to transform these into more general patterns (Bryman, 2016). This is all the more problematic because of the additional influence of contextual factors. Still, our findings may deserve further research to discover such patterns by analysing and comparing different participatory practices.

A second limitation is that the empirical distinction between conceptual rationales was sometimes hard to make, as these are mostly neither strictly defined nor operationalised. For instance, in the Research trajectory, it was sometimes hard to distinguish the substantive from the instrumental rationale by the criteria we chose. Therefore, future empirical studies may benefit from a further operationalisation of the rationales.

A final limitation is that we only interviewed patients who were directly involved in the participatory practice of Q‐support. This sample did not allow us to establish whether the broader transformative effects—as reported by our respondents—were indeed experienced by Q fever patients not participating in Q‐support. Studying such public effects might have added value in future research.

## 5 CONCLUSION

We conclude that invited participation may gain in clarity by making explicit the rationales for participation. If placed at the centre of attention, and given a leading role in the design of the dimensions of the participatory process, this ‘intentionality’ may contribute to reaching the full potential of invited patient participation. That is, such ‘clarity through specificity’ provides opportunities for managing conflicts between rationales and for understanding the possible interactions of rationales and dimensions, as the building blocks of participation, through more thorough evaluation studies. By making explicit what is often left implicit, our approach may help to prevent invited participation from becoming mere window‐dressing.

## CONFLICT OF INTEREST

At the time of the study, CW held a position in the participatory practice case studied. She was the chair of the Scientific Committee that designed the patient participation with respect to the assessment of the research proposals submitted to the organization. In that role, she also acted as a respondent in the semi‐structured interviews for the study.

## AUTHOR CONTRIBUTION

JH and CW conceptualised, and JH designed the study. KK collected the data. KK, JH and LR analysed and interpreted the data. KK drafted the manuscript. JH, CW and LR reviewed the first draft. KK and JH revised the manuscript. All authors approved the final manuscript.

## Data Availability

The data that support the findings of this study are available from the corresponding author upon reasonable request.
